# Aloe-inspired eco-friendly synthesis of Ag/ZnO heterostructures: boosting photocatalytic potential

**DOI:** 10.1038/s41598-024-61466-9

**Published:** 2024-06-03

**Authors:** Nawal Ansar, Wajeehah Shahid, Muhammad Atif Irshad, Samiah Shahid, Rab Nawaz, Ali Irfan, Muhammad Iftikhar Khan, Aamal A. Al-Mutairi, Maria Khizar, Sami A. Al-Hussain, Sana Ullah, Magdi E. A. Zaki

**Affiliations:** 1 Department of Physics, The University of Lahore, Lahore, 54000 Pakistan; 2 Department of Environmental Sciences, The University of Lahore, Lahore, 54000 Pakistan; 3 Institute of Molecular and Biology and Biotechnology, The University of Lahore, Lahore, 54000 Pakistan; 4 Faculty of Engineering and Quantity Surveying, INTI International University, 71800 Nilai, Negeri Sembilan Malaysia; 5 Department of Chemistry, Government College University Faisalabada, Faisalabad, Pakistan; 6 Department of Chemistry, College of Science, Imam Mohammad Ibn Saud Islamic University (IMSIU), 11623 Riyadh, Saudi Arabia

**Keywords:** Environmental sciences, Chemistry, Materials science, Nanoscience and technology, Physics

## Abstract

The current research focuses on the development of Ag–ZnO heterostructures through a “bottom-up” approach involving the assembly and extraction of *Aloe barbadensis* Miller gel. These heterostructures composed of metals/semiconductor oxide display distinct and notable optical, electrical, magnetic, and chemical properties that are not found in single constituents and also exhibit photocatalytic applications. These synthesized heterostructures were characterized by XRD, FTIR, SEM, and UV–visible spectroscopy. The high peak intensity of the Ag/ZnO composite shows the high crystallinity. The presence of Ag–O, Zn–O, and O–H bonding is verified using FTIR analysis. SEM analysis indicated the formation of spherical shapes of Ag/ZnO heterostructures. The Zn, O, and Ag elements are further confirmed by EDX analysis. Ag–ZnO heterostructures exhibited excellent photocatalytic activity and stability against the degradation of tubantin red 8BL dye under visible light irradiation.

## Introduction

Silver and zinc oxide nanostructures are the most widely used metallic and metallic oxide nanostructures. Zinc oxide nanostructures have gained significant attention in research due to their increased industrial potential as well as their high piezoelectric properties, large binding energy, and low toxicity. Additionally, owing to their exceptional anti-oxidant activity, zinc oxide nanostructures are among the most widespread inorganic nanostructures. Zinc oxide nanostructures, an n-type semiconductor with a direct band gap of 3.37 eV^1^, have been generally utilized in water decontamination photocatalysis owing to their notable photocatalytic features such as their physically and chemically stable structure, low affordability, biocompatibility, strong oxidizing ability, high photosensitivity, and high availability^2^. On the other hand, due to electron–hole recombination, which results in reduced active species on the photocatalyst surface, the wide band gap in ZnO led to non-optimal photocatalytic activity^3^. Therefore, to improve ZnO photocatalytic activity, it is essential to prevent electron–hole recombination through appropriate modifications. Additionally, they offer potential for environmental and biological applications because of their non-toxic nature. ZnO nanostructures are widely used in several applicable areas, including energy storage^4^, dye-sensitized solar cells^5^, nanosensors^6^, optoelectronic devices^7^, nano-electronics^8^, gas sensors^9^, photocatalysts^10^, spintronics^11^, and biomedical treatment^12^. ZnO nanostructures have been employed as semiconductors in the fabrication of microelectronics and pollutant degradation. ZnO nanostructures are also suitable for UV screening applications due to their greater chemical stability and low toxicity^13^.

Silver nanostructures are attracting much interest due to their carrier properties, which include anti-microbial^14^, anti-inflammatory^15^, anticancer drugs^16^, and antioxidant activities^17^. Unique biological, chemical, and physical properties of silver nanostructures among metallic nanostructures are advantageous, reserving their potentiality in industrial applications. Ag nanostructures have an exceptional wide-spectrum of anti-fungal, anti-bacterial, and anti-oxidant activities that are commonly used in several electronic devices such as washing machines, televisions, and refrigerators, as well as for water and air purification, food packaging, clothing, cosmetics, and medicine^18–20^. Moreover, the morphological features of synthesized Ag nanostructures are significantly affected by their anti-oxidant activity^21,22^. In order to exploit the anti-oxidant activity in the fabrication of innovative products, a variety of plant extracts have been utilized to fabricate Ag nanostructures in various shapes and sizes^23^. Ag nanostructures, including those treated with plant extracts, have gained considerable attention, especially owing to their well-documented anti-oxidant and anti-bacterial activities^24–27^.

*Aloe vera* is systematically identified as *Aloe barbadensis* Miller. The Liliaceae family includes *Aloe vera*^28^. The succulent plant *Aloe vera*, which has thorns on its branches and waxy coatings, grows readily in dry environments^29,30^. Three layers comprise *Aloe vera* leaf: the exterior portion, which is dense and protective, includes a pretty good amount of cellulose; the central portion is composed of primary flavanone (aloin A and B); and the interior portion contains a fresh gel that is made of sugars, vitamins (A, B, C, and E), proteins, anthraquinones, amino acids, and acetylated glucomannan^31–33^. *Aloe vera* leaf consists of latex, gel, and rind. Since ancient times, *Aloe vera* gel has been widely employed as a traditional medicine to heal different skin ailments, inflammations, and minor burns, as well as as a source of vitamins and minerals^34^. The green rind is a consequence of gel extraction, which involves strong active substances that can be implemented to manufacture metal and metallic oxide nanoparticles containing polysaccharides, proteins, tannins, flavonoids, and glycosides such as aloin. The plant *Aloe vera* and its numerous components, such as leaf extract and gel, have revealed several beneficial uses that facilitate an extensive variety of applications. Acetic acid, citric acid, ascorbic acid, pectin, lignin, polyphenols, hemicellulose, and flavonoids are the primary biomolecules that exist in *Aloe vera* leaves and are capable of being used as stabilizing and reducing agents in the green fabrication of metallic nanoparticles^35^. A clear mucilaginous constituent named *Aloe vera* gel is generally composed of water, fibres, and chemicals that help maintain moisture^36^.

The current study focuses on the synthesis of Ag/ZnO heterostructures for biomedical applications via *Aloe vera* gel extract. We produce the photocatalytic activity of Ag/ZnO heterostructures through the use of *Aloe vera* extracts. To this end, we take advantage of the fact that *Aloe vera* gel includes a wide range of different chemicals utilizable as an agent that act on the surface and hinder nucleus accumulation by decreasing overall surface energy. Based on the findings of the present study, we illustrate that the synthesized Ag/ZnO heterostructures are eco-friendly and can be employed as a potent for efficient photocatalytic activity. Finally, we explain how the current work has been successfully utilized in a variety of environmental domains.

## Materials and methods

Analytical-grade chemicals and precursors needed in experiments are purchased from Sigma Aldrich Company and used without further purification because all chemicals are 99% pure. Among these chemicals are powdered forms of silver nitrate (AgNO_3_), zinc nitrate hexahydrate Zn (NO_3_)_2_.6H_2_O, and sodium hydroxide (NaOH). To synthesize the Ag/ZnO nanocomposites as starting materials, deionized water, ethanol, and fresh *Aloe vera* leaves have been utilized. Ag/ZnO composites/nanocomposites were synthesized via ex-situ techniques.

### Gel extract preparation

*Aloe vera* fresh leaves were collected from plants and washed completely with water. Then, we dried them in the air for about twenty minutes to prepare the aqueous extract of *Aloe vera* leaves. The gel was isolated from the leaves. 400 g of gel was divided into chunks and crushed with the help of a pestle and mortar. The gel was blended with a comparable quantity of distilled water and dried for 40 min at 85 °C in an oven. The prepared extract was stored in a refrigerator at room temperature for further experiments and then filtered using filter paper.

### Synthesis of silver (Ag) nanostructures

An aqueous solution of AgNO_3_ was prepared by mixing 0.5 M of AgNO_3_ in 40 mL of distilled water, and the solution was magnetically stirred for 30 min at 85 °C. 40 mL of plant extract, *Aloe vera*, was gradually added to the AgNO_3_ solution, and the above mixture was kept under magnetic stirring for about 1 h. Then, sonicate the abovementioned mixture for 2 h. The final solution was then transferred into a Teflon-lined, sealed vessel of 100 ml capacity under 150 °C conditions for 6 h. The centrifugation technique is used to separate a dark grey precipitation that was continually rinsed with ethanol and deionized water after being dried in an oven for 12 h at 80 °C.

### Synthesis of zinc oxide (ZnO) nanostructures

To prepare zinc oxide nanostructures, 0.5 M zinc nitrate hexahydrate Zn (NO_3_)_2_.6H_2_O was dispersed in 20 mL of deionized water and continued on magnetic stirring at 85 °C for 20 min. We added 20 mL of plant extract, *Aloe vera*, to the above mixture. Through this procedure, a solution of 0.5 M NaOH in 20 mL of deionized water was made. We further added the NaOH solution drop-wise to the above mixture and left it on magnetic stirring for 1 h. The abovementioned solution was sonicated for 1 h. The solution was transferred to a 100 mL coated Teflon-lined jar and heated at 150 °C for 6 h. The pale-white precipitation was gathered via centrifugation, repetitively scrubbed with ethanol and deionized water, and formerly dried for 12 h in an oven at 80 °C.

### Ex-situ synthesis of Ag/ZnO heterostructures

Ag/ZnO heterostructures have been synthesized using an ex-situ approach. In this approach, previously-made nanostructures of ZnO and Ag have been utilized. Initially, in order to prepare the Ag/ZnO heterostructures with a molar ratio of 1:1, 0.2 g of ZnO was mixed in 20 mL of ethanol, and 0.2 g of Ag was mixed in 20 mL of ethanol, and both mixtures were combined together for 30 min with continuous stirring. Then add a drop-wise Ag solution to the ZnO solution and maintain magnetic stirring for 1 h. We then sonicated the aforementioned mixture for 1 h. Later on, the mixture was transferred into the centrifuge tube to gather the particles. Then it was dried in an oven for 6 h at about 80 °C. A dark grey precipitation was formed after grinding.

### Photocatalytic activity

The photocatalytic activity was investigated using the Tubantin red 8BL dye. The study was carried out in the presence of a sun simulator named Abet Technologies Sunlight TM solar simulator. Prior to being employed in subsequent degradation experiments, each of these catalysts (ZnO and Ag/ZnO) was disinfected, implementing a range of cleaning and centrifugation methods. In order to estimate the catalyst's reusability and stability, three cycles were performed. The following equation is used to examine the degree of degradation: the pseudo-first-order process is a catalytic decolorization of dye solution^37,38^.

1 $${\text{Degradation }}\left( \% \right) = \left( {\frac{{C_{0} - C_{t} }}{{C_{0} }}} \right) \times 100\%$$

Here $C_{0}$ is the initial concentration, and here is the concentration after a particular amount of time. The degradation was calculated with the help of 50 mL of aqueous Tubantin red 8BL dye (10 ppm) for 10 min, 20 min, 30 min, 40 min, 50 min, and 60 min in the light and for 30 min in the dark, respectively. All the samples were achieved with 0.015 g of the catalyst’s standard pH. The photocatalytic breakdown mechanism was utilized to determine the absorption peak characteristics of tubantin red 8BL dye. In the absence of catalyst, there was particularly no degradation.

## Results and Discussion

### XRD Analysis

**Figure 1:**
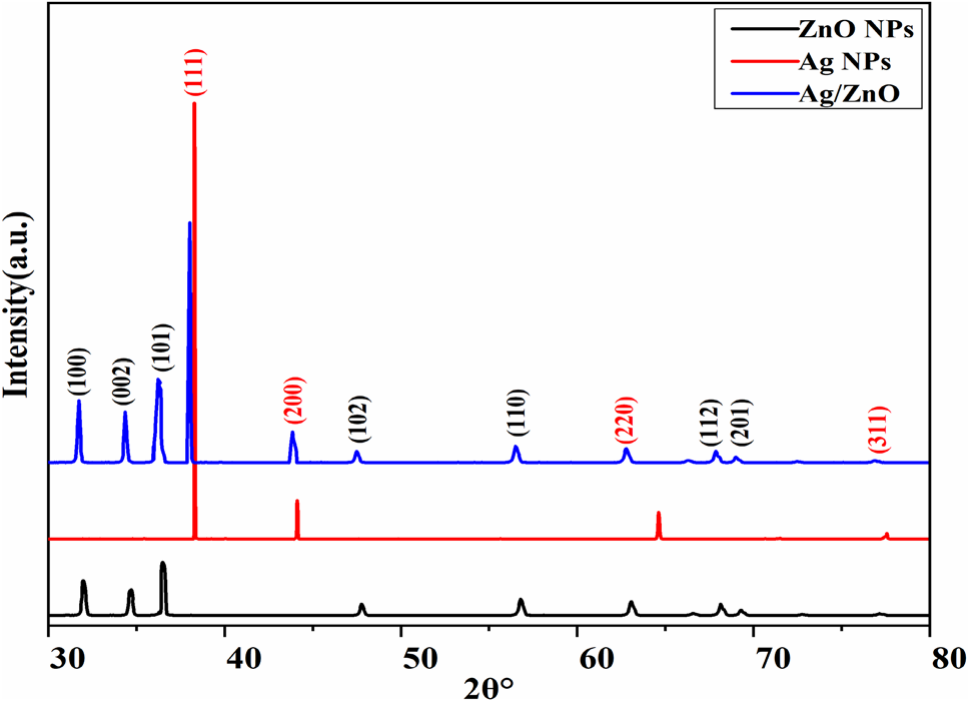
XRD spectrum of Ag, ZnO and Ag/ZnO heterostructures.

X-ray diffraction was used to analyze the crystalline nature of the prepared heterostructures. Figure 1 illustrates the XRD pattern of prepared Ag/ZnO heterostructures using *Aloe vera* gel extract. Sharp diffraction patterns were observed at 2$\theta$ = $31^\circ$, $34^\circ$, $36^\circ$, $47^\circ$, $56^\circ$, $67^\circ$, and $69^\circ$, which were ascribed to diffraction planes having indices (100), (002), (101), (102), (110), (112), and (201), respectively, that resemble the hexagonal crystal structure of ZnO nanostructures (JCPDS-files: PDF#36-1451). No other impurity peaks were found in the diffraction pattern, showing that all the precursors have been totally transformed into nanostructures, and therefore the resulting ZnO nanostructures are extremely crystalline in nature. The XRD pattern shows that only a single phase exists in ZnO nanostructures with the space group (P63mc). The crystalline character of the prepared specimen presents the sharp and broad nature of diffraction patterns. The comparatively higher intensity (101) peak implies a preferred alignment and anisotropic growth of crystallites. It is obvious from the results of XRD analysis that there were characteristic diffraction patterns with a distinct value of sharp peaks at 2$\theta$ = $38^\circ$ and $43^\circ$ attained, which correspond to the diffraction forms of (111) and (200) orientations. Based on standard diffraction data card no. (PDF#04-0783), some additional peaks occurred at $62^\circ$ and $77^\circ$ were compatible with diffraction planes (220) and (311), respectively. The examined Bragg’s reflection correlating to (111) and (200) may be recorded the prediction of the face-centered cubic crystal structure of Ag nanostructures. This pattern affirmed that there are no additional phases other than the FCC structure of metallic Ag. The confirmation of phase purity and synthesis of elemental silver were clearly investigated for consistency with reference data^39^. Furthermore, the presence of Ag in the ZnO lattice is verified by two additional peaks that have been identified as (111) and (200). Similar findings have been reported for Ag/ZnO heterostructures^39–45^.

### FTIR analysis

**Figure 2:**
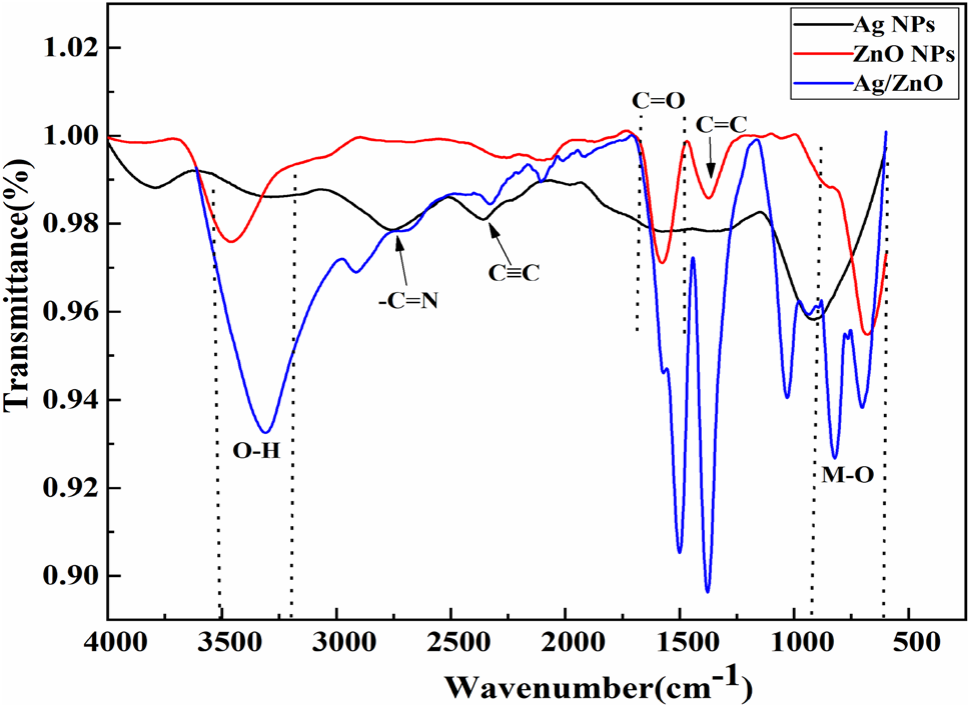
Fourier transmission spectra of Ag, ZnO and Ag/ZnO heterostructures.

The purity of samples and identification of functional groups were observed in the range of 500–4000 cm^−1^ using FTIR analysis. Figure 2 depicts the FTIR spectra of Ag nanostructures, ZnO nanostructures, and Ag/ZnO heterostructures synthesized using *Aloe vera* gel. The strong absorption band at 3200–3500 cm^−1^ correlates with the overlaying of the stretching vibrations of O–H in the phenolic group and N–H in the amine group^46^. With the transformation of Ag^+^ ions into Ag and Zn^+2^ ions into ZnO, the O–H groups in phenols behave as reducing agents ^46^. The peaks in the range of 2700–cm^−1^ are also linked with amide groups, which contain amino acids and proteins present in the gel extract. The stretching vibrations of alkanes, alkenes, and alkynes are attributed to the bands at 2089 cm^−1^, 1360 cm^−1^, and 2370 cm^−1^, respectively^47^. The bands in the region of 1760–1520 cm^−1^ are ascribed to C=O carboxylic acids. Furthermore, the small vibrations in the region of 1476–1019 cm^−1^ indicate the presence of C–N groups of aromatic and aliphatic amines, –C–O– or –C–O–C-stretching vibrations, and aliphatic fluoro compounds (C–F) functional groups, which signifies the accessibility of flavonoids and reducing sugars^48^. Broader peaks observed below 1000 cm^−1^ indicate the occurrence of metal oxide vibrations (M–O). The sharp and dominant peaks were observed at 823.9 cm^−1^ and 712.2 cm^−1^, which might be attributed to Ag–O and Zn–O bonding. The high band intensity of Ag/ZnO heterostructures usually signifies an increase in functional groups connected to the molecular bond, while the shifting of absorption bands designates an alteration in the hybridization state or arrangement of electrons in the molecular bonds. The absorption peak shows the interaction of H-bond formation with the O–H functional groups in Ag/ZnO heterostructures. FTIR measurements illustrate the carboxyl, alcoholic, and Ag–O and Zn–O functional groups, which confirm the fabrication of Ag/ZnO heterostructures^41,46^.

### SEM analysis

**Figure 3:**
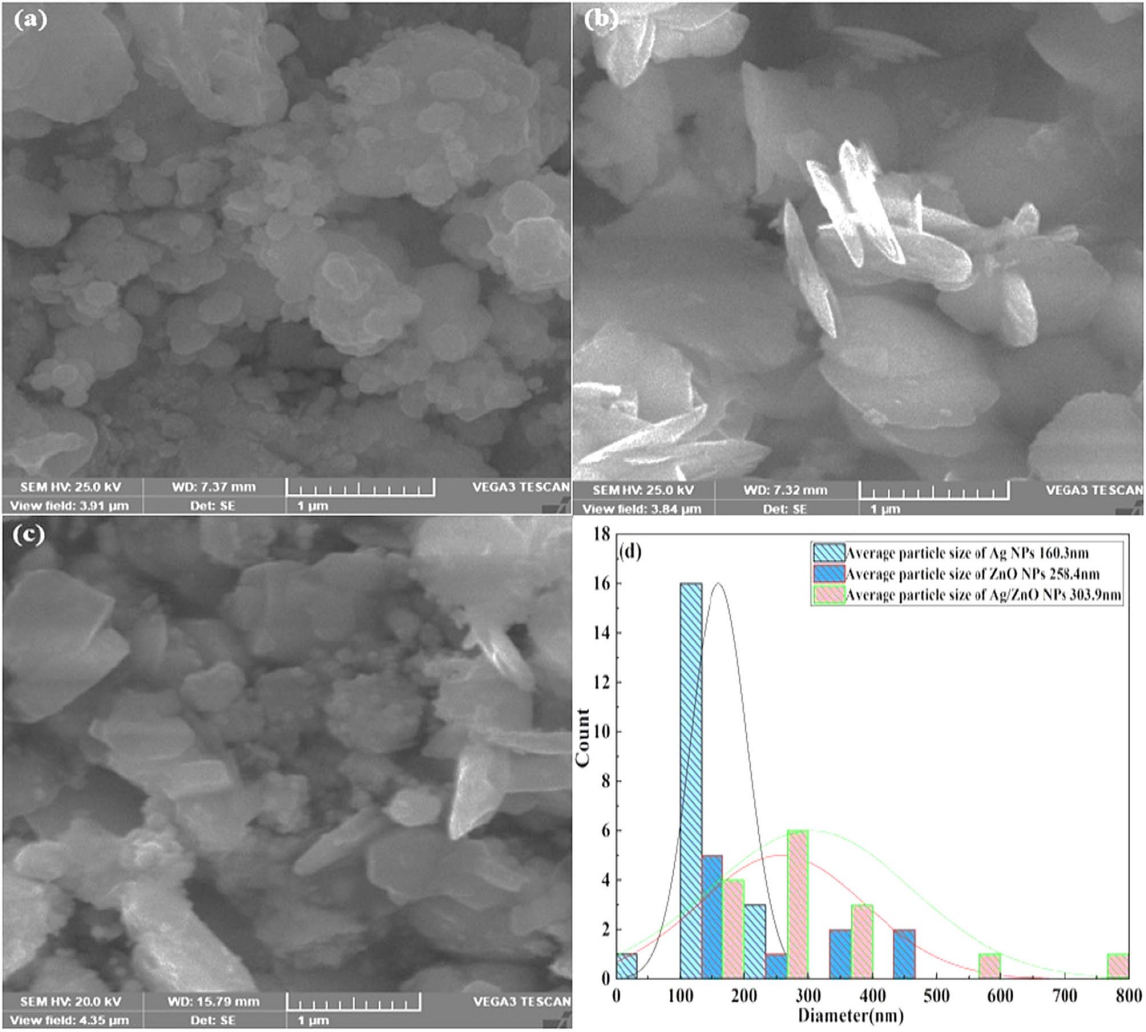
SEM Analysis of (**a**) Ag (**b**) ZnO and (**c**) Ag/ZnO heterostructures, (**d**) Average particle size distribution.

The morphological properties of prepared nanostructures were investigated via the SEM technique. The growth mechanism, shape, and surface morphology of Ag nanostructures, ZnO nanostructures, and Ag/ZnO heterostructures are illustrated in Fig. 3a–c. It is examined that *Aloe vera* gel extract acts as capping and reducing agents, leading to the fabrication of Ag/ZnO heterostructures in various shapes. The randomly oriented and densely packed spherical shapes of silver nanostructures are clearly observed using SEM analysis in Fig. 3a. The collected clusters that are distributed over the surface with a huge random empty space were displayed in the SEM image^49^. The nanostructure agglomeration shows that they were in direct contact and indicates the stability of the formation of Ag. The majority of nanostructures exhibit a spherical shape, though some nanostructures demonstrate cubic shapes that are not precisely determined. The synthesized ZnO nanostructure’s morphology and the micrographs are presented in Fig. 3b. The formation of uniformly dispersed ZnO nanosheets with definite particle boundaries was clearly observed in Fig. 3b. The 2D nanosheets are interconnected with each other to form a flower configuration. The micrographs indicated the three-dimensional nanoflower-like structures of ZnO nanostructures. Regarding the *Aloe vera* gel extract, more homogeneity of nanoflowers was examined in ZnO nanostructures^50^. The SEM image revealed that the shape of ZnO nanostructures is somehow nano needle-like morphology^51^. Figure 3c depicts the SEM analysis of Ag/ZnO heterostructures. The micrograph indicates that the morphology of heterostructures is irregular and non-uniform in shape. The arrangement of particles looks like randomness in the form of clusters. This morphology implies that the composition might not be homogenous and the synthesized procedure might not have been uniformly optimized. It is observed that some spherical shapes are present due to Ag nanostructures on the surface of ZnO^40^. However, a small amount of Ag nanostructures appears to be comparatively tiny in size, whereas a huge amount of Ag agglomerates at the surface of porous and dispersed ZnO^39^. The formation of some irregular nanosheets was due to the presence of Ag nanostructures covering the surface of ZnO nanostructures. This agglomeration is generated because of the densification produced by the small space among the particles. The particles in Ag/ZnO heterostructures are conspicuously more agglomerated, with the creation of tiny particles over bigger clusters, which might be attributed to the fabrication of Ag nanostructures^44,52^. Furthermore, different morphologies might be attributed to the gel extract of the bioactive components. The presence of different shapes of nanostructures could be produced due to the bio-component nature of the extract^53,54^.

Figure 3d represented the average particle size distribution of prepared nanostructures. The average size was 160.3 nm for Ag nanoparticles, 258.4 nm for ZnO, and 303.9 nm for Ag/ZnO. From this, we can clearly confirm the fact that average particle size was increased in composite as compared to Ag and ZnO.

### EDX analysis

**Figure 4:**
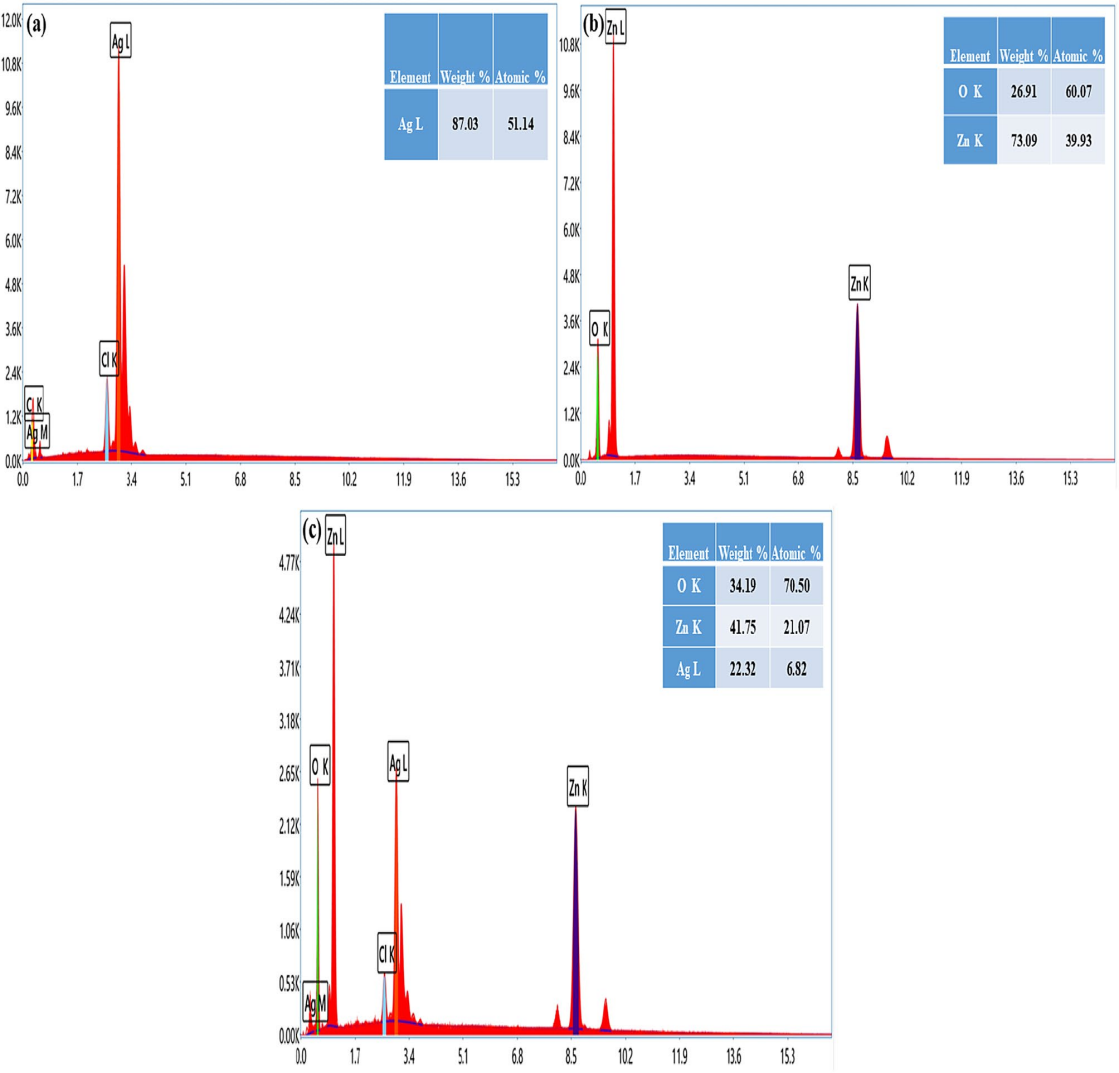
EDX spectra of (**a**) Ag, (**b**) ZnO and (**c**) Ag/ZnO heterostructures.

The most effective characterization technique for identifying the synthesis of Ag, ZnO, and Ag/ZnO nanostructures is EDX analysis. EDX measurements may also be employed to investigate the elemental composition. Figure 4a–c describes the EDX spectrum of Ag, ZnO, and Ag/ZnO heterostructures using *Aloe vera* gel. The fabrication of Ag nanostructures was confirmed by the strong peak for metallic silver at 3 keV observed in the EDX spectrum in Fig. 4a. The existence of silver elements is represented by a line in the EDX plot. Therefore, the formation of Ag nanostructures from *Aloe vera* gel can be confirmed. The total composition of silver is 87.0% (wt%), which is not found in previous literature. In order to observe the chemical composition of ZnO nanostructures, the graph depicts the two sharp and strong peaks associated with zinc and one distinct peak of the oxygen element. Figure 4b reveals the significant peaks of zinc and oxygen, which verify that these are the two major components in nanostructures. The produced sample contains no impurities of other elements, which strongly confirmed the formation of high-purity ZnO nanostructures. The EDX spectrum of Ag/ZnO heterostructures indicates the specific peaks of Zn, O, and Ag. The distributions of elements in the analysis presented peaks of Zn, O, and Ag with atomic percentages of 21.07%, 70.50%, and 6.82%, respectively. While weight percentages are taken into consideration, the exposed peaks of 41.75% for Zn, 34.19% for O, and 22.32% for Ag are represented in Fig. 4c. For zinc, there were two prominent signals near 1.5 keV and 8.5 keV. The only significant peak of the oxygen element was generated at 0.6 keV, whereas in the case of silver, two moderate peaks were observed at approximately 0.2 keV and 3.0 keV. Some minor peaks of C and Cl were examined in the EDX spectra. These elements were obtained from the biomolecules that were bound to the surfaces of Ag nanostructures and Ag/ZnO heterostructures in *Aloe vera* gel^45,55^.

### UV analysis

The optical absorption properties of ZnO, and Ag/ZnO heterostructures prepared via *Aloe vera* gel extract in the range of 200–800 nm were examined. The equation representing the Tauc relationship^56^ was utilized for calculating the prepared samples optical band gap values:

2 $$\alpha h\nu = A \left( {h\nu - E_{g} } \right)^{n} .$$

**Figure 5:**
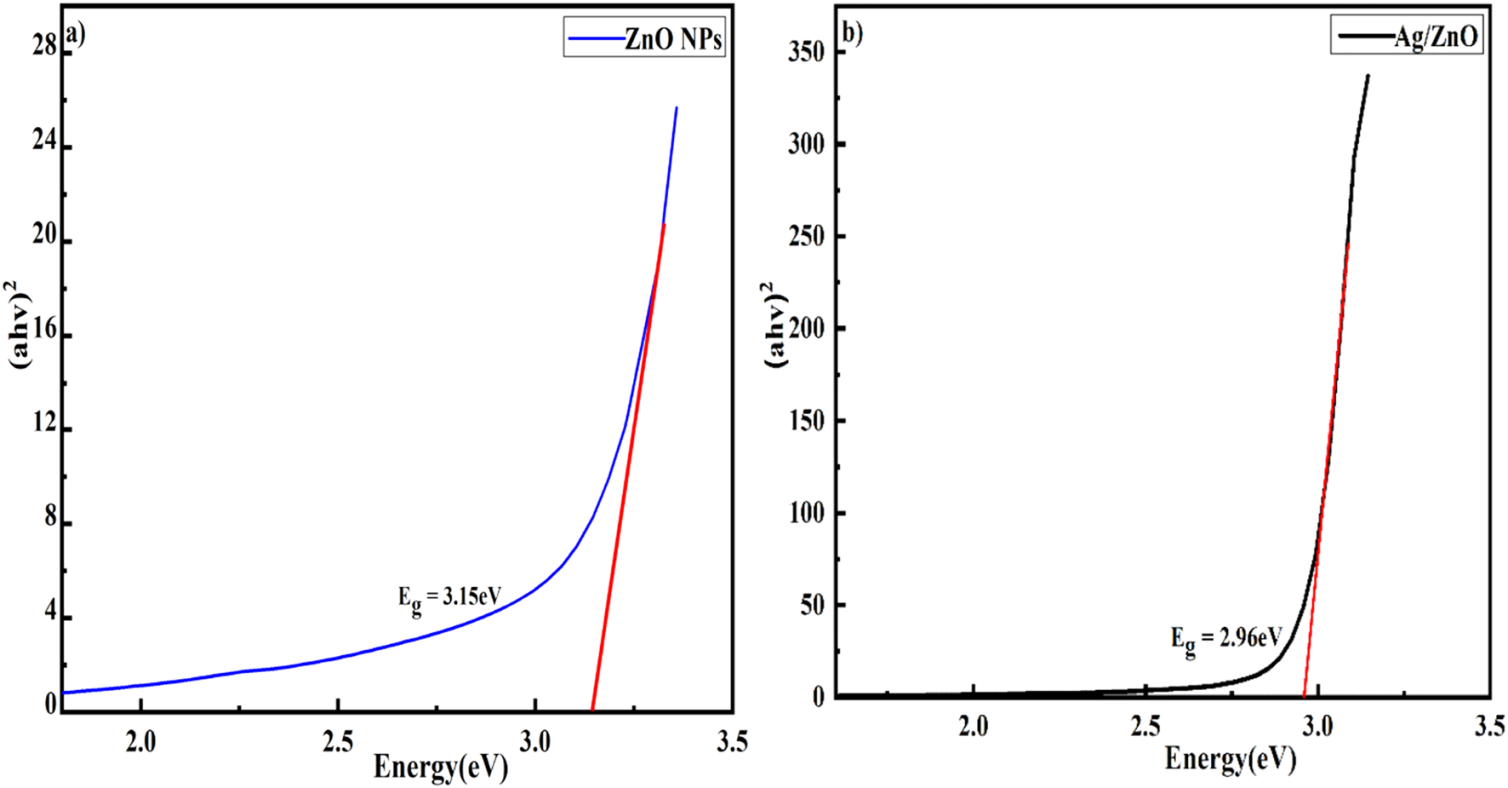
Tauc Plot of (**a**) ZnO and (**b**) Ag/ZnO heterostructures.

In this equation, “E_g_” signifies the optical bandgap energy, “*h*” is the Plank’s constant, “$\nu$” represents frequency, “$\alpha$” symbolizes the absorption coefficient and “A” is a constant that depends on transition probability^57,58^. Here, n relies on electrons transition and the value of n = 1/2 belongs to direct electrons transition with a direct optical band gap. Figure 5a,b exhibit a plot between $\alpha h\nu^{2}$ and $E_{g}$ that determines the optical band gap energy of prepared nanocomposites. This Tauc plot indicated a band gap of 3.15 eV and 2.96 eV for ZnO and Ag/ZnO heterostructures, respectively. The decrease in energy band gap in Ag/ZnO heterostructures was observed when Ag is embedded into the ZnO. This might be due to n-type conductivity of metallic ions. The oxygen vacancies generated in ZnO, which may capture impurities and possible chemical reactions originated among ZnO and Ag surface could also be involved in narrowing the energy band gap. This contributes the more oxygen vacancies, whereas high electron density was produced in ZnO. Ag^+^ replaces the Zn^2+^ sites, leading to increasing electron densities and vacancies due to variation in ionic radius which lowers the Fermi level and brings about narrowing the band gap.

### Tubantin red 8BL photodegradation

**Figure 6:**
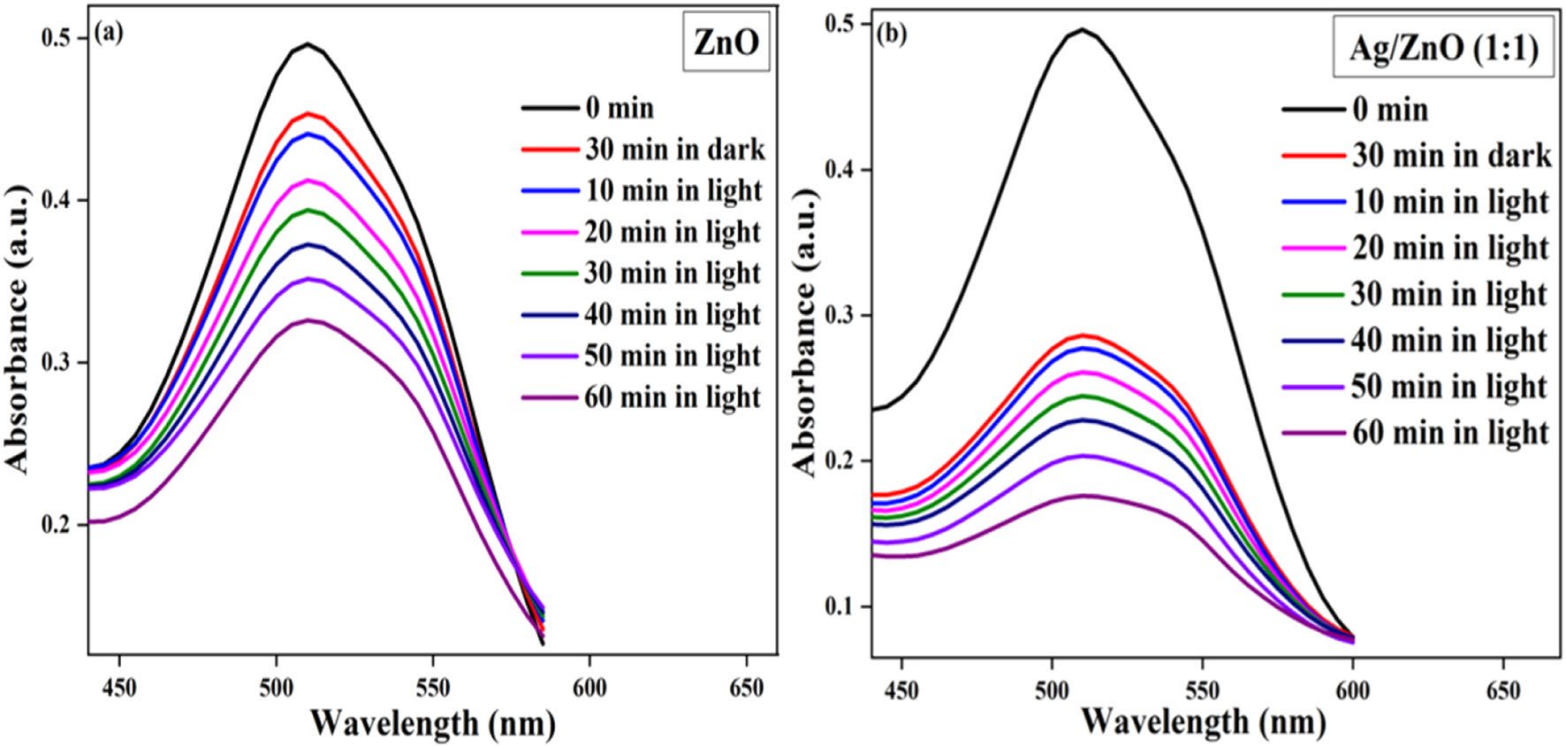
Uv–visible absorption spectrum of the Tubantin red 8BL dye with catalyst (**a**) ZnO and (**b**) Ag/ZnO (1:1) heterostructures.

The photocatalytic activity of fabricated ZnO and Ag/ZnO heterostructures was studied by examining the degradation of tubantin red 8BL dye using solar light irradiation. The absorption band of tubantin red 8BL was positioned at 510 nm and reduced subsequently with increasing irradiation time. The photodegradation and absorption bands versus time of Tubantin red 8BL dye acting as a photocatalyst ZnO and Ag/ZnO (1:1) heterostructures at a concentration of 0.015 g are represented in Fig. 6a,b. The photodegradation method of Tubantin red 8BL dye displays the characteristic band at 510 nm, which is a distinctive standard indicator, and the degradation of synthesized ZnO and Ag/ZnO (1:1) heterostructures was also performed under dark conditions. A catalyst’s degradation of tubantin red 8BL dye was revealed to cause the decrease in concentration over time.

**Figure 7:**
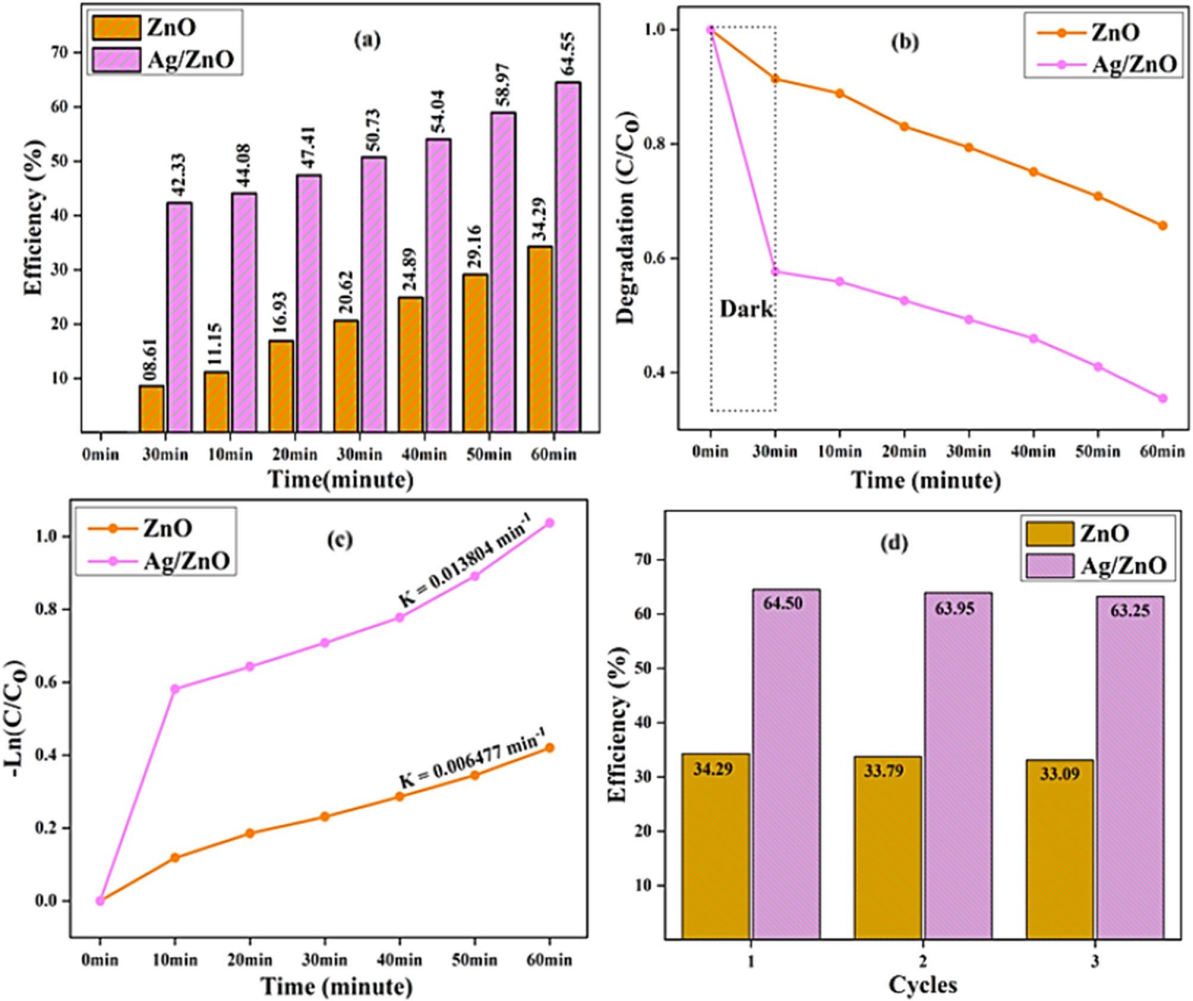
(**a**) Irradiation time verses efficiency (%); (**b**) degradation ($C/C_{o}$) over time; (**c**)—Ln ($C/C_{o}$) versus irradiation time; (**d**) % efficiency verses cycle graphs of ZnO and Ag/ZnO (1:1) heterostructures.

Figure describes the initial absorption band and the absorption spectrum for the photocatalytic activity of ZnO and Ag/ZnO (1:1) heterostructures after 30 min in the dark and 10 min, 20 min, 30 min, 40 min, 50 min, and 60 min in the light. Figure 7a indicates the correlation among efficiency (%) and time for the ZnO and Ag/ZnO (1:1) heterostructures. The measured efficiency values of ZnO nanostructures in the presence of light 11.15%, 16.93%, 20.62%, 24.89%, 29.16% and 34.29%; and for Ag/ZnO (1:1) heterostructures efficiency values are 44.08%, 47.41%, 50.73%, 54.04%, 58.97% and 64.55% correspondingly. The efficiency values of ZnO and Ag/ZnO (1:1) heterostructures in 30 min dark conditions is analogously observed around 08.61% and 42.33%, respectively.

The connection between degradation rate ($C/C_{0}$) and irradiation time (min) is explained in Fig. 7b. The degradation rate of Ag/ZnO (1:1) heterostructures are 0.5593, 0.5245, 0.4898, 0.4608, 0.4085 and 0.3535 and ZnO nanostructures are 0.8869, 0.8291, 0.7899, 0.7507, 0.7072, and 0.6551 respectively after 10, 20, 30, 40, 50 and 60 min in the presence of light. Likewise after 30 min under dark conditions, the degradation rates of pure and nanocomposites are 0.9116 and 0.57338 respectively. Figure 7c examines the graph of Ln ($C/C_{0}$) values over time for the Ag/ZnO (1:1) are 0.5791, 0.6427, 0.7063, 0.7768, 0.8895 and 1.0354 and for the ZnO nanostructures are 0.1114, 0.1868, 0.2313, 0.2854, 0.3418 and 0.4195 respectively. The rate constant values of Ag/ZnO (1:1) heterostructures and ZnO nanostructures are 0.013804 and 0.006477 min^−1^ accordingly, demonstrating the pseudo-1st order kinetics and has significant photocatalytic activity. The stability of Ag/ZnO (1:1) and ZnO synthesized heterostructures has been confirmed using three cycles as represented in Fig. 7d. The degradation characteristics and stability of generated heterostructures were obviously visible after being subjected to solar radiation after 60 min.

## Conclusions

In the current work, Ag/ZnO heterostructures were successfully synthesized using the hydrothermal method via *Aloe vera* gel extract. The Ag/ZnO heterostructures are preferentially oriented along the (111) plane, which was verified by using XRD analysis. FTIR measurements exhibited that the absorption bands demonstrate the formation of H-bonds and their interaction with O–H functional groups in Ag/ZnO heterostructures. The SEM analysis reveals the existence of both ZnO and Ag in spherical shapes that could be produced due to the bio-component nature of the gel extract. The EDX spectrum designates the weight percentages of Zn, O, and Ag as 41.75%, 34.19%, and 22.32%, respectively. The energy band gap (E_g_) observed in Ag/ZnO heterostructures is 2.96 eV. Ag/ZnO exhibited a maximum photodegradation efficiency of 64.55%, demonstrating that it is a promising material for excellent photocatalytic degradation. The aforementioned findings imply that the synthesized Ag/ZnO heterostructures could be helpful in numerous fields, including the elimination of harmful organic dyes from wastewater, and can be used in various biomedical domains.

## Data Availability

Data will be provided on request.

## References

[1] Gea S (2022). Facile synthesis of ZnO–Ag nanocomposite supported by graphene oxide with stabilised band-gap and wider visible-light region for photocatalyst application. JMRT.

[2] Xue B, Zou Y (2018). High photocatalytic activity of ZnO–graphene composite. J. Colloid Interface Sci..

[3] Yang SJ (2011). MOF-derived ZnO and ZnO@ C composites with high photocatalytic activity and adsorption capacity. J. Hazard. Mater..

[4] Chakraborty S, Mary NL (2020). Biocompatible supercapacitor electrodes using green synthesised ZnO/Polymer nanocomposites for efficient energy storage applications. J. Energy Storage.

[5] Suliman AE (2007). Preparation of ZnO nanoparticles and nanosheets and their application to dye-sensitized solar cells. Sol. Energy Mater..

[6] Que M (2021). Progress in ZnO nanosensors. Sensors.

[7] Xu L (2018). Characterization of Ag-doped ZnO thin film for its potential applications in optoelectronic devices. Optik.

[8] Alrajhi AH, Ahmed NM, Shanker U (2023). Green synthesis of zinc oxide nanoparticles using salvia officinalis extract. Handbook of Green and Sustainable Nanotechnology: Fundamentals, Developments and Applications.

[9] Galstyan V (2015). Nanostructured ZnO chemical gas sensors. Ceram. Int..

[10] Noman MT (2019). One-pot sonochemical synthesis of ZnO nanoparticles for photocatalytic applications, modelling and optimization. J. Mater..

[11] Sharma DK (2022). A review on ZnO: Fundamental properties and applications. Mater. Today.

[12] Islam F (2022). Exploring the journey of zinc oxide nanoparticles (ZnO-NPs) toward biomedical applications. Materials.

[13] Kaur R (2021). Lignin-based metal oxide nanocomposites for UV protection applications: A review. J. Clean. Prod..

[14] Ismail M (2016). Plant mediated green synthesis of anti-microbial silver nanoparticles—A review on recent trends. Rev. Nanosci. Nanotechnol..

[15] Zhang X-F (2016). Silver nanoparticles: Synthesis, characterization, properties, applications, and therapeutic approaches. Int. J. Mol. Sci..

[16] Hussein HA (2022). MA Abdullah, Novel drug delivery systems based on silver nanoparticles, hyaluronic acid, lipid nanoparticles and liposomes for cancer treatment. Appl. Nanosci..

[17] Ameen F (2023). Anti-oxidant, anti-fungal and cytotoxic effects of silver nanoparticles synthesized using marine fungus *Cladosporium halotolerans*. Appl. Nanosci..

[18] Alaallah NJ (2023). Eco-friendly approach for silver nanoparticles synthesis from lemon extract and their anti-oxidant, anti-bacterial, and anti-cancer activities. J. Turk. chem. soc..

[19] Kumar S (2021). Plant extract mediated silver nanoparticles and their applications as antimicrobials and in sustainable food packaging: A state-of-the-art review. Trends Food Sci..

[20] Paiva-Santos AC (2021). Plant-mediated green synthesis of metal-based nanoparticles for dermopharmaceutical and cosmetic applications. Int. J. Pharm..

[21] Ahmadi O (2018). Eco-friendly microwave-enhanced green synthesis of silver nanoparticles using *Aloe vera* leaf extract and their physico-chemical and antibacterial studies. Green Process. Synth..

[22] Mohammadlou M, Maghsoudi H, Jafarizadeh-Malmiri H (2016). A review on green silver nanoparticles based on plants: Synthesis, potential applications and eco-friendly approach. Int. Food Res. J..

[23] Abdel-Aziz MS (2014). Antioxidant and antibacterial activity of silver nanoparticles biosynthesized using *Chenopodium murale* leaf extract. J. Saudi Chem. Soc..

[24] Kim M (2019). Green-synthesis of anisotropic peptone-silver nanoparticles and its potential application as anti-bacterial agent. Polym. J..

[25] Baruah K (2021). *Ocimum sanctum* mediated green synthesis of silver nanoparticles: A biophysical study towards lysozyme binding and anti-bacterial activity. J. Mol. Liq..

[26] Melkamu WW, Bitew LTJH (2021). Green synthesis of silver nanoparticles using *Hagenia abyssinica* (Bruce) JF Gmel plant leaf extract and their antibacterial and anti-oxidant activities. J. Mol. Liq..

[27] Mohanta YK (2017). Phyto-assisted synthesis of bio-functionalised silver nanoparticles and their potential anti-oxidant, anti-microbial and wound healing activities. IET Nanobiotechnol..

[28] Burange PJ (2021). Synthesis of silver nanoparticles by using *Aloe vera* and *Thuja orientalis* leaves extract and their biological activity: A comprehensive review. BNRC.

[29] Nandal U, Bhardwaj RL (2012). *Aloe vera*: A valuable wonder plant for food, medicine and cosmetic use-a review. Int J Pharm Sci Rev Res..

[30] Chow JT-N (2005). Chemical characterization of the immunomodulating polysaccharide of *Aloe vera* L. Carbohydr. Res..

[31] Reynolds T, Dweck AC (1999). *Aloe vera* leaf gel: A review update. J. Ethnopharmacol..

[32] Anju T (2021). Green synthesis of silver nanoparticles from *Aloe vera* leaf extract and its antimicrobial activity. Mater. Today Proc..

[33] Zaidan M (2005). In vitro screening of five local medicinal plants for antibacterial activity using disc diffusion method. Trop. Biomed..

[34] Rathor N (2012). Acute effect of *Aloe vera* gel extract on experimental models of pain. Inflammation.

[35] Zhang Y (2013). Biosynthesis of silver nanoparticles at room temperature using aqueous aloe leaf extract and antibacterial properties. Colloids Surf. A Physicochem. Eng. Asp..

[36] Kumar D (2012). A review of immunomodulators in the Indian traditional health care system. J. Microbiol..

[37] Shahid W, Idrees F, Iqbal MA, Tariq MU, Shahid S, Choi JR (2022). Ex situ synthesis and characterizations of MoS_2_/WO_3_ heterostructures for efficient photocatalytic degradation of RhB. Nanomaterials.

[38] Ullah S, Shahid W, Shahid S, Khan MI, Ansar N, Khizar M (2023). Advancing photocatalysis: Innovative approaches using novel V_2_O_5_/ZnO nanocomposites for efficient photocatalytic degradation of tubantin red. J. Saudi. Chem. Soc..

[39] Slathia S, Gupta T, Chauhan R (2021). Green synthesis of Ag–ZnO nanocomposite using *Azadirachta indica* leaf extract exhibiting excellent optical and electrical properties. Matter. Phys. Rev. B Condens..

[40] Jadhav P (2020). Green AgNPs decorated ZnO nanocomposites for dye degradation and antimicrobial applications. Eng. Sci..

[41] Panchal P (2020). Biogenic mediated Ag/ZnO nanocomposites for photocatalytic and antibacterial activities towards disinfection of water. J. Colloid Sci..

[42] Nagaraju G (2017). Electrochemical heavy metal detection, photocatalytic, photoluminescence, biodiesel production and antibacterial activities of Ag–ZnO nanomaterial. Mater. Res. Bull..

[43] Jobe MC (2022). Biosynthesis of zinc oxide and silver/zinc oxide nanoparticles from *Urginea epigea* for antibacterial and antioxidant applications. Heliyon.

[44] Alharthi FA (2020). Facile one-pot green synthesis of Ag–ZnO Nanocomposites using potato peeland their Ag concentration dependent photocatalytic properties. Sci. Rep..

[45] Mtavangu SG (2022). In situ facile green synthesis of Ag–ZnO nanocomposites using *Tetradenia riperia* leaf extract and its antimicrobial efficacy on water disinfection. Sci. Rep..

[46] Das B (2016). Understanding the antifungal mechanism of Ag@ ZnO core-shell nanocomposites against *Candida krusei*. Sci. Rep..

[47] Sohal JK (2019). Determination of antioxidant potential of biochemically synthesized silver nanoparticles using *Aloe vera* gel extract. Plant Sci. Today..

[48] PP VJB (2020). In vitro biocompatibility and antimicrobial activities of zinc oxide nanoparticles (ZnO NPs) prepared by chemical and green synthetic route—a comparative study. BNSC.

[49] Gecer EN (2022). Green synthesis of silver nanoparticles from *Echinacea purpurea* (L.) Moench with antioxidant profile. JPST.

[50] Mohan S (2020). Hydrothermal synthesis and characterization of zinc oxide nanoparticles of various shapes under different reaction conditions. Nano. Exp..

[51] Venkataraju JL (2014). Synthesis, characterization and evaluation of antimicrobial activity of zinc oxide nanoparticles. J. Biochem. Tech..

[52] Khan MS (2020). Ultrasound-assisted green synthesis of ag-decorated ZnO nanoparticles Using *Excoecaria agallocha* leaf extract and evaluation of their photocatalytic and biological activity. ChemistrySelect..

[53] SJ LJR (2023). Green synthesis, characterizations, and antibacterial activity of silver nanoparticles from *Themeda quadrivalvis*, in conjugation with macrolide antibiotics against respiratory pathogens. RAMS.

[54] Medda S (2015). Biosynthesis of silver nanoparticles from *Aloe vera* leaf extract and antifungal activity against Rhizopus sp. and Aspergillus sp. Appl. Nanosci..

[55] Yuharmon N (2018). Scanning electron microscopy of soxhlet extracted *Aloe vera* gel for electrolyte application. JPCS.

[56] Feng Y (2015). Can Tauc plot extrapolation be used for direct-band-gap semiconductor nanocrystals?. J. Appl. Phys..

[57] Yusof KN (2018). Parkia speciosa as reduction agent in green synthesis silver nanoparticles. ChemistrySelect.

[58] Maraj M (2021). Mo-doped CuO nanomaterial for photocatalytic degradation of water pollutants under visible light. Catalysts.

